# Supplementary material for: DxFormer: a decoupled automatic diagnostic system based on decoder–encoder transformer with dense symptom representations
Source: Bioinformatics. 2022 Nov 21;39(1):btac744. doi: 10.1093/bioinformatics/btac744 (PMC9825744; doi:10.1093/bioinformatics/btac744)
Supplement: btac744_Supplementary_Data [file btac744_supplementary_data.pdf]

Supplementary Material for A Benchmark for Automatic Medical Consultation System: Frameworks, Tasks and Datasets

November 14, 2022

Contents

1 Defects of Traditional RL Methods 1

2 Algorithm Details and Advantages 1

3 Case Study 2

4 The Effect of Reward Setting in DxFormer 4

5 Author Contribution 4

1 Defects of Traditional RL Methods

We believe that traditional RL approaches do not perform well in symptom recall for two reasons: 1) In traditional RL framework, the action spaces of symptom inquiry and disease diagnosis are coupled (or joint), from where the agent selects an action at each step. Once the agent's chosen action is a disease, the entire session is terminated. The agent may be "afraid" of asking wrong symptoms that gets negative reward, and make a diagnosis eagerly, thus converging to a local optimal solution. Under the setting of traditional RL approaches, the number of interactive turns is determined by the reward and training settings, and it is not controllable; 2) In most past studies, symptoms are one-hot encoded as separate categories and simple MLP framework is utilized for policy learning. This simple setting is difficult to handle high-dimensional action space. Moreover, as the input features of the disease classifier, the increase of the number of symptoms will lead to a more sparse feature space, which may become intractable for the disease classifier.

2 Algorithm Details and Advantages

To clarify the working details of DxFormer more clearly, we show pseudo code of the process of automatic diagnosis in Algorithm 1.

At the beginning, the explicit symptoms are known. In each turn of interaction of agent, the decoder is used for symptom inquiry, that is, select a symptom based on known symptoms, and ask whether the patient has the symptom. The union of explicit symptoms and collected symptoms will be used as the input of the encoder, and the probability distribution of disease prediction is

computed. If the maximum probability exceeds the set threshold, or exceeds the maximum number of inquiry rounds, the diagnosis process is ended; otherwise, the next turn will be conducted.

This decoupling method can overcome the shortcomings of the traditional RL model. The advantages of DxFormer are: 1) Symptom query and disease diagnosis are decoupled, and they can be independently optimized without being affected; 2) In each step, symptom query and disease diagnosis will be both executed, and whether more symptom features are needed will be determined according to the confidence level of disease diagnosis; 3) Symptoms are regarded as tokens in natural language, which are densely represented rather than one-hot encoded; 4) The transition from symptom query to disease diagnosis is parametrically determined by stopping criteria, and the number of interactive rounds can be flexibly controlled.

Algorithm 1 Pseudo code of DxFormer during inference.

S_{exp}: Explicit symptoms.
$S_{clt} = \varnothing$: Collected Symptoms.
Enc: Trained encoder, Dec: Trained decoder.
P: Patient Simulator.
$T_{max} = 10, \epsilon = 0.95, i = 0$.
while $i < T_{max}$ **do**
    $sx = Dec(S_{exp}, S_{clt})$ ▷ Select a symptom from the set of all symptoms.
    $attr = P(sx)$ ▷ Ask the patient if they have the symptom, where $attr \in \{\text{POS}, \text{NEG}, \text{UNK}\}$.
    if $attr \neq$ UNK **then**
        $S_{clt} = S_{clt} \cup \{sx, attr\}$
    end if
    $d = Enc(S_{exp}, S_{clt})$ ▷ Compute the probability distribution of the disease based on the collected symptoms.
    if $max(d) \geq \epsilon$ **then**
        break
    end if
    $i = i + 1$
end while

3 Case Study

We also show a case analysis in Table 1, which comes from a case in the MZ-4 test set. The patient has 1 explicit symptom. The agent has completed the correct diagnosis through 7 rounds of symptom inquiry.

We can find some logic here. At the beginning, the agent think that the patient has the highest probability (62.3%) of suffering from *Infant Dyspepsia*. After knowing that the patient has the symptoms of diarrhea, the probability of *Infant Diarrhea* became the highest (75.9%). Then the agent continuously asks about 4 related symptoms, including loose stool, watery stool, cough and fever, but all receives unknown feedback. Finally, when it is known that the patient has abdominal pain and does not fart, the final diagnosis result of the agent is adjusted to *Infant Dyspepsia* (99.6%).

DxFormer found 3 out of 4 implicit symptoms, reaching a symptom recall of 75% in this case, and the effect of crying symptom on diagnosis is not significant. In an ideal state, DxFormer is

Case study of automatic diagnosis on MZ-4 test set based on DxFormer.

Explicit symptoms: vomit (POS)

Implicit symptoms: crying(POS), diarrhea (POS), abdominal pain (POS), fart (NEG)

Disease: Infantile Dyspepsia

**Step**	**Action**	**Action Type**	**Feedback**	**Confidence (Threshold: 99%)**
0	-	-	-	infantile dyspepsia (62.3%)
1	**diarrhea**	symptom inquiry	POS	infantile diarrhea (75.9%)
2	loose stool	symptom inquiry	UNK	infantile diarrhea (75.9%)
3	watery stool	symptom inquiry	UNK	infantile diarrhea (75.9%)
4	cough	symptom inquiry	UNK	infantile diarrhea (75.9%)
5	fever	symptom inquiry	UNK	infantile diarrhea (75.9%)
6	**fart**	symptom inquiry	NEG	infantile dyspepsia (92.6%)
7	**abdominal pain**	symptom inquiry	POS	infantile dyspepsia (99.6%)
8	infantile dyspepsia	disease diagnosis	-	-

Collected symptoms: vomit (POS), diarrhea (POS), fart(NEG), abdominal pain (POS)

Symptom recall: 75%. (3 of the 4 implicit symptoms are found.)

expected to accurately predict the possible symptoms based on the known information of symptoms, and enhance the diagnostic accuracy through richer symptom features.

4 The Effect of Reward Setting in DxFormer

In order to explore the impact of reward settings in reinforcement learning, we conduct two additional groups of experiments. One group is to fix priority reward, and adjust the value of negative reward in ground reward; The other group is to fix the ground reward and adjust the negative reward value in the priority Reward. We present the experimental results in Table 2. Note that the stopping criterion threshold are uniformly set to 1.0 for all the experiments in Table 2.

It can be seen that the negative ground Reward parameter is sensitive. A proper punishment can help to improve the symptom recall, but it must not be excessive, otherwise the symptom inquiry module can not be effectively trained. On the other hand, the priority reward parameter is relatively gentle, which has little impact on the symptom recall, as long as the negative priority reward is not set too small. The purpose of setting Priority Reward is to accelerate the convergence of the model.

5 Author Contribution

Wei Chen contributed the main ideas of the proposed method, conducted a large number of experiments, and wrote this manuscript;
Cheng Zhong contributed to the reproduction of baseline models, and helped the experiment with constructive discussions and involved in drafting the manuscript;
Jiajie Peng helped to design the whole picture of the research and contributed to the paper writing and modification;
Zhongyu Wei monitored the whole research project and provided guidance to the discussion of the creation of the model architecture, the design of the experiments, result analysis and manuscript writing.

Effect of reward setting on symptom recall.

Priority Reward		Ground Reward		SX-Rec		
Pos	Neg	Pos	Neg	Dxy	MZ-4	MZ-10
1	-1	2.5	0	0.732	0.545	0.591
		2.5	-0.5	**0.749**	**0.556**	0.597
		2.5	-1	0.678	0.552	**0.602**
		2.5	-1.5	0.246	0.514	0.591
		2.5	-2	0.022	0.272	0.500
		2.5	-2.5	0.016	0.249	0.414
		2.5	-3	0.011	0.141	0.338
1	0	2.5	-0.5	0.743	**0.556**	0.601
1	-0.5			**0.743**	0.554	**0.603**
1	-1			0.738	0.553	0.600
1	-1.5			0.727	0.550	0.595
1	-2			0.727	0.551	0.598
